# Anticancer and antiradical scavenging activity of *Ageratum conyzoides* L. (Asteraceae)

**DOI:** 10.4103/0973-1296.59968

**Published:** 2010-02-13

**Authors:** A. H. Adebayo, N. H. Tan, A. A. Akindahunsi, G. Z. Zeng, Y. M. Zhang

**Affiliations:** 1 *State Key Laboratory of Phytochemistry and Plant Resources in West China, Kunming Institute of Botany, Chinese Academy of Sciences, Kunming 650204, China*; 2 *Department of Biological Sciences, College of Science and Technology, Covenant University, PMB 1023, Ota, Ogun State, Nigeria*; 3 *Department of Biochemistry, Federal University of Technology, Akure, Nigeria*

**Keywords:** *Ageratum conyzoides*, anticancer, antiradical scavenging activity, cytotoxicity

## Abstract

*Ageratum conyzoides* has been used in folklore for the treatment of a wide range of diseases. In the present investigation, the *in vitro* activity of ethanol, petroleum ether, ethylacetate, butanol, and water extracts of *A. conyzoides* were screened in some cancer cell lines using the sulphorhodamine B (SRB) assay. These cell lines include: Human non-small cell lung carcinoma (A-549), human colon adenocarcinoma (HT-29), human gastric carcinoma (SGC-7901), human golima (U-251), human breast carcinoma (MDA-MB-231), human prostate carcinoma (DU-145), human hepatic carcinoma (BEL-7402), and mouse leukemia (P-388) cancer cell lines. Furthermore, kaempferol was isolated from the ethylacetate extract and the structure was elucidated by nuclear magnetic resonance (NMR) and mass spectroscopy. The effect of DPPH antiradical activity on the extracts and kaempferol was also determined. The results showed that ethylacetate extract exhibited the highest cytotoxic activity on A-549 and P-388 cancer cells with IC_50_ values of 0.68 and 0.0003 μg/ml, respectively. Kaempferol isolated from the ethylacetate extract of *A. conyzoides* rapidly scavenged DPPH at a concentration of 130.07 ± 17.36 g/kg. The result therefore showed that *A. conyzoides* possessed anticancer and antiradical properties.

## INTRODUCTION

Cancer is a major public health problem in both developed and developing countries. It was estimated that there were 10.9 million new cases, 6.7 million deaths, and 24.6 million persons living with cancer around the world in 2002.[1,2] In the US, cancer is the second leading cause of death,[3] where one in four deaths is due to cancer.[2] Since 1990, there has been a 22% increase in cancer incidence and mortality with the four most frequent cancers being lung, breast, colorectal, and stomach.[4,5] Medicinal plants have played important roles in the last five decades in the treatment of cancer and most new clinical applications of plant secondary metabolites and their derivatives have been applied towards combating cancer. *Ageratum conyzoides* L. belongs to the family and tribe of Asteraceae and Eupatoriae, respectively. It is native to Central America, Caribbean, Florida (USA), Southeast Asia, South China, India, Nigeria, Australia, and South America.[6,7] It is traditionally called “ufu opioko” and “otogo” by the Igedes in Benue state, Nigeria.[8] In Southwestern Nigeria, it is known as “Imí esú”.[9] *A. conyzoides* has been used in folklore for the treatment of fever, pneumonia, cold, rheumatism, spasm, headache, and curing wounds.[10,11] Its gastroprotective,[11] antibacterial,[12] anti-inflammatory, antianalgesic, antipyretic,[6] anticoccidial,[13] and anticonvulsant[14] properties have been reported. However, to the best of our knowledge, the *in vitro* anticancer activity of this plant has never been evaluated and published. Natural products from plants can be another potent source for the discovery of anticancer and antioxidant agents. In this article, we describe the *in vitro* anticancer activity of ethanol, petroleum ether, ethylacetate, butanol, and water extracts of *A. conyzoides* against some cancer cell lines: Human non-small cell lung (A-549), human gastric (SGC-7901), human colon (HT-29), human golima (U-251), human breast carcinoma (MDA-MB-231), human prostate carcinoma (DU-145), human hepatic carcinoma (BEL-7402), and mouse leukemia (P-388) cancer cell lines as well as the antiradical activity of the aforementioned extracts using the DPPH method.

## MATERIALS AND METHODS

### Plant material

The leaves of *Ageratum conyzoides* L. were obtained from the campus of Covenant University, Ota, Ogun State, Nigeria, in December, 2007. The plant was authenticated at the Department of Pharmacognosy, University of Lagos, Lagos, Nigeria and a voucher specimen (PCGH 436) was deposited in the Herbarium for reference purpose.

### Reagents and cancer cell lines

All extraction reagents used such as ethanol, petroleum ether, ethylacetate, and n- butanol were of analytical reagent grade. Trypsin, trizima base, sulforhodamine B (SRB), trichloroacetic acid, RPMI-1640 culture medium, and trypan blue solutions were purchased from Sigma Chemicals Co., Ltd. (St. Louis, MO, USA). 1,1-diphenyl -2- picryl hydrazyl (DPPH) free radical reagent was bought from Tokyo Chemical Industry company (TCI), Japan. The purity of methanol reagent used from Yuwang Group, Shandong, China was 99.9%. Human non-small cell lung carcinoma (A-549), human colon adenocarcinoma (HT-29), human gastric carcinoma (SGC-7901), human golima (U-251), human breast carcinoma (MDA-MB-231), human prostate carcinoma (DU-145), human hepatic carcinoma (BEL-7402), and mouse leukemia (P-388) cancer cell lines were obtained from Shanghai Institute of Materia Medica, Chinese Academy of Sciences (Shanghai, China) and the Cell Culture Centre of Institute of Basic Medical Sciences, Chinese Academy of Medical Sciences (Peking, China).

### Extraction and fractionation of the plant

The procedure described by[15] was adopted. The leaves of *A. conyzoides* were collected and air-dried in the laboratory for two weeks after which they were blended into fine powder. One hundred grams were extracted with 95% ethanol. Evaporation of the extract in a rotatory evaporator (Buchi 461, Switzerland) at 40°C yielded an ethanol extract (32 g). The residue (14 g) was dissolved in deionized water (100 mL) and successively extracted with petroleum ether (0.5 L × 4), ethylacetate (0.5L × 5), and n-butanol (0.5 L × 5) solvents. The petroleum ether, ethylacetate, n-butanol, and water fractions yielded 1.1, 0.5, 1.1, and 1.6 g, respectively. Each fraction was evaporated under reduced pressure and subsequently screened for anticancer and antiradical activities.

### Purification, isolation, and structure elucidation of compound

The ethylacetate extract (173 g) was fractionated by column chromatography (CC) (1.4 kg silica gel, mesh 100-200; CHCl_3_: MeOH 9:1, 7:3, 1:1, 3:7 and 1:4). Fractions 3 and 4 were respectively separated by CC (1. sephadex LH-20 CHCl_3_: MeOH 2:1; 2. Pet. ether: Acetone 2:1,) and afforded kaempferol (5.0 mg). 3,5,7,4′-tetrahydroxyflavone, kaempferol, yellow powder, C_15_H_10_O_6_, ^1^H-NMR (400 MHz, CD_3_OD), δ_H_: 6.17 (1H, d, *J* = 1.4 Hz, H-6), 6.38 (1H, s, H-8), 8.07 (1H, d, *J* = 8.7 Hz, H-2), 6.89 (1H, d, *J* = 8.7 Hz, H-3), 6.89 (1H, d, *J* = 8.7 Hz, H-5), 8.07(1H, d, *J* = 8.7 Hz, H-6).^13^C-NMR (100 MHz, CD_3_OD), δc: 148.0(s, C-2), 137.1(s, C-3), 177.4 (s, C-4), 104.5 (s, C-4a), 162.5 (s, C-5), 99.2 (d, C-6), 165.6 (s, C-7), 94.4 (d, C-8), 160.5 (s, C-8a), 123.7 (s, C-1), 130.7 (d, C-2), 116.3 (d, C-3), 158.2 (s, C-4), 116.2 (d, C-5), 130.7 (d, C-6). EI-MS *m/z* (%): 286 [M]^+^ (100), 285 (35), 258 (14), 229 (14), 213 (12), 121 (43), 69 (21). EI-MS was recorded on a VG Auto Spec-3000 spectrometer (Britain). Proton Nuclear Magnetic Resonance (^1^H-NMR) and Carbon-13 (^13^C)-NMR spectra were recorded on Bruker AM-400 and DRX-500 spectrometers (Rheinstteten, Germany) with tetramethylsilane (TMS) as internal standard. Column chromatography was carried out over silica gel (200-300 mesh, Qingdao Marine Chemical Inc., China) and sephadex LH-20 (25-100 μm, Pharmacia Fine Chemical Co., Ltd., Sweden), respectively.

### Cancer cell growth inhibition assay

The procedures described by[16,17] were followed. Briefly, the sulphorhodamine B (SRB) assay was adopted for a quant measurement of cell growth and viability. Cells were seeded in 96-well microtiter plates at 3000-7000 cells per well. After 24 hour, extracts were added to a final concentration of 10 μg/ml and incubated for 48 hour. Cells were then fixed by the addition of 50% ice-cold Cl_3_CCOOH and then left at 4°C for 1 hour. After washing, air-drying and staining was done for 15 minutes with 100 μl 0.4% SRB in 1% glacial AcOH; excessive dye was removed by washing with 1% glacial AcOH. The absorbance values of resuspended SRB in 10 mM Tris buffer was read at 560 nm on a microplate spectrophotometer (SPECTRA MAX 340, USA); further assessment was carried out with at least four serially diluted concentrations (dilution ratio 1:2) with sample inhibition of 50% or more to obtain the IC_50_ value. The IC_50_ value was calculated from the following formular: $\log_{10}({\mathrm{Ic}}_{50})=\frac{\log_{10}C_{L}(I_{H}-50)+\log_{10}C_{H}(50-I_{L})}{I_{H}-I_{L}}$ ${\mathrm{IC}}_{50}=10\log_{10}({\mathrm{IC}}_{50})$

Where:

I_H_: I% above 50%

I_L_: I% below 50%

C_H_: High drug concentration

C_L_: Low drug concentration

### DPPH antiradical assay

The effects of 1,1-diphenyl-2-picrylhydrazyl (DPPH) antiradical or antioxidant potentials of the extracts were determined according to the procedures described by[18] with slight modifications. Aliquots of 5 μL of dimethyl sulphoxide (DMSO) solutions each containing extract of four replicates were added to 195 μL of pure methanol solution of DPPH (0.025 g/L). Absorbance at 515 nm against a blank of methanol without DPPH was determined after 30 minutes of incubation at room temperature in a microplate spectrophotometer (SPECTRA MAX 340, USA). Six serially diluted concentrations of samples with initial % activity of 50 or more were prepared and using the above procedure, absorbance of each sample concentration against a methanol blank was read. A graph of % activity against concentration was plotted for each sample. The EC_50_ value, which is the amount of antioxidant necessary to decrease the initial DPPH concentration by 50%, was determined.

## RESULTS

### Characterization of compound

**Figure 1:**
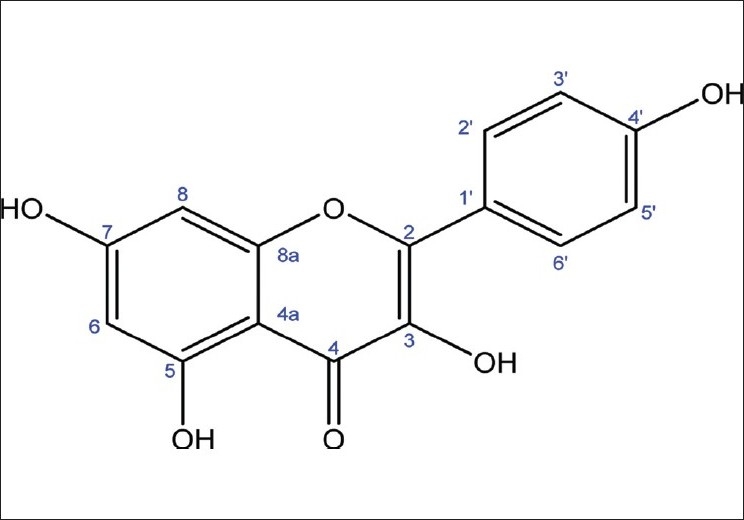
Structure of kaempferol

The compound [Figure 1] obtained was yellow and its molecular weight as determined by Fast Atom Bombardment Mass Spectroscopy (FAB-MS) was 286. From the ^1^H-NMR, ^13^C-NMR, and Distortionless Enhancement by Polarization Transfer (DEPT) spectra, the molecular formula, C_15_H_10_O_6_ was derived. From the spectra data, it was found that the structure of the compound belongs to flavonoid group and when the structure was compared with established literature data, it was identified as 3,5,7,4′-tetrahydroxyflavone (kaempferol).[19,20]

### DPPH antiradical activity

**Figure 2:**
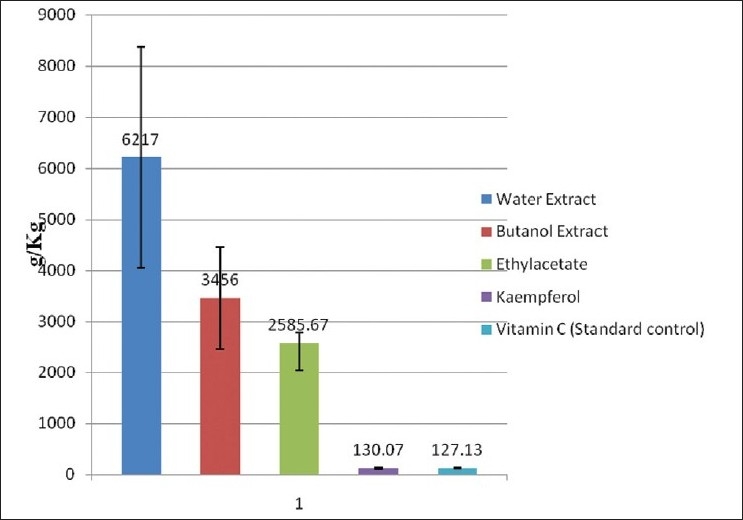
DPPH scavenging activity of extracts and compounds from *Ageratum conyzoides*

The preliminary result of the effect of extracts of *A. conyzoides* on the DPPH scavenging activity showed that the water extract, n-butanol extract, and ethylacetate extract have % activity of 87.80, 85.40, 72.56, and 60.87, respectively, while the petroleum ether extract had a % activity of 7.84 [Table 1]. EC_50_ value is the effective concentration required to scavenge 50% of the DPPH radical and was extrapolated from a linear graph. Kaempferol showed an EC_50_ value of 130.07 ± 17.36 g/ kg and was highly comparable to the standard control (vitamin C) with an EC_50_ value of 127.13 ± 8.56 g/kg [Figure 2].

**Table 1:** Preliminary investigation of DPPH scavenging activity of the leaf extracts of *Ageratum conyzoides*

Extracts	DPPH assay (mg/mL)	Activity (%)
Petroleum ether	0.31 ± 0.013	7.84
Ethylacetate	0.09 ± 0.010	72.56*
n-butanol	0.05 ± 0.015	85.40*
Water	0.04 ± 0.016	87.80*
Ethanol	0.13 ± 0.016	60.87*
Ascorbic acid (vit C)	0.01 ± 0.001	96.87*

Tabulated values are mean ± standard deviation of three replicates.

* I % > 50 of samples which were re-evaluated to determine their EC_50_ values

### Anticancer activity

From the preliminary anticancer screening, the petroleum ether extract of *A. conyzoides* exhibited a significant inhibition (I > 50%) on SGC-7901, HT-29, and P-388 cancer cell lines [Tables 2 and 3]. Similarly, the ethylacetate extract showed a significant inhibition on A-549, SGC-7901, HT-29, P-388, MDA-MB-231, and DU-145 cancer cell lines. Furthermore, ethanol extract had a significant inhibitory activity on HT-29 and P-388 cancer cell lines. n-Butanol and water extracts showed no significant inhibition (I < 50%) on any of the cancer cell lines. Table 4 shows the IC_50_ values of ethanol, petroleum ether, and ethylacetate extracts. The ethanol extract showed an IC_50_ value of 1.73 μg/ml in P-388 cell line, while petroleum ether extract had IC_50_ values of 14.06, 13.77, and 0.71 μg/ ml in A-549, SGC-7901, and P-388 cells, respectively. Similarly, the ethylacetate extract showed IC_50_ values of 0.68, 9.97, 14.88, and 0.0003 μg/ml in A-549, DU-145, SGC-7901, and P-388 cells, respectively.

**Table 2:** Treatment of the leaf extracts of *Ageratum conyzoides* with human non-small cell lung cancer cell line (A 549), human gastric cancer cell line (SGC 7901), human colon cancer cell line (HT-29), and human golima cancer cell line (U-251)

Extracts	A 549 (mg/mL)	SGC 7901 (mg/mL)	HT 29 (mg/mL)	U-251 (mg/mL)
Control	1.60 ± 0.08	1.60 ± 0.08	0.85 ± 0.12	0.45 ± 0.05
Petroleum ether	0.44 ± 0.02 (39.42)	0.77 ± 0.02 (52.02)*	0.60 ± 0.05 (53.30)*	0.52 ± 0.02 (−17.55)
Ethylacetate	0.25 ± 0.02 (66.01)*	0.57 ± 0.02 (64.37)*	0.29 ± 0.04 (66.24)*	0.43 ± 0.03 (2.73)
n-Butanol	0.99 ± 0.05 (−35.09)	1.61 ± 0.01 (−3.44)	0.87 ± 0.02 (0.22)	0.64 ± 0.02 (−42.85)
Water	0.90 ± 0.06 (−23.66)	1.68 ± 0.04 (−5.09)	1.19 ± 0.09 (7.93)	0.65 ± 0.02 (−46.45)
Ethanol	0.56 ± 0.05 (23.94)	0.86 ± 0.02 (26.71)	0.37 ± 0.02 (60.27)*	0.44 ± 0.06 (13.65)

Tabulated values are Mean ± Standard deviation of three replicates. Values in parenthesis indicate percentage inhibition (I %)

* I % > 50 of samples which were further evaluated to determine their IC_50_

**Table 3:** Treatment of the leaf extracts of *Ageratum conyzoides* with human breast carcinoma cell line (MDA-MB-231), human prostate carcinoma cell line (DU-145), human hepatic carcinoma cell line (BEL-7402), and mouse leukemia cell line (*P* - 388)

Extracts	MDA-MB 231 cells (μg/mL)	DU-145 cells (μg/mL)	BEL-7402 cells (μg/mL)	P-388 cells (μg/mL)
Control	0.76 ± 0.16	0.38 ± 0.02	0.48 ± 0.06	0.74 ± 0.08
Petroleum ether	0.50 ± 0.10 (34.376)	0.26 ± 0.01 (31.26)	0.39 ± 0.01 (19.86)	0.031 ± 0.003 (95.88)*
Ethylacetate	0.25 ± 0.01 (67.57)*	0.07 ± 0.01 (81.06)*	0.27 ± 0.02 (44.01)	0.04 ± 0.003 (94.62)*
n-Butanol	0.70 ± 0.01 (8.70)	0.38 ± 0.02 (−0.26)	0.47 ± 0.03 (3.13)	0.29 ± 0.06 (23.01)
Water	0.84 ± 0.15 (−9.53)	0.41 ± 0.01 (−6.53)	0.52 ± 0.01 (−6.30)	0.78 ± 0.10 (−4.75)
Ethanol	0.54 ± 0.13 (29.52)	0.34 ± 0.01 (11.41)	0.45 ± 0.01 (6.30)	0.171 ± 0.03 (76.93)*

Tabulated values are Mean ± Standard deviation of three replicates. Values in parenthesis indicate percentage inhibition (I %)

* I % > 50 of samples which were further evaluated to determine their IC_50_

**Table 4:** Extracts of *Ageratum conyzoides* showing IC_50_ values of some cancer cell lines

Cells	Ethanol (μg/ml)	Petroleum ether extract (μg/ml)	Ethylacetate extract (μg/ml)	n-Butanol extract (μg/ml)	Water extract (μg/ml)	Taxol (μg/ml)	HCPT (μg/ml)
A-549	-	14.06	0.68	-	-	0.02	0.031
DU-145	-	-	9.90	-	-	0.04	-
MDA-MB-231	-	-	-	-	-	-	3.14
BEL-7402	-	-	-	-	-	0.58	-
SGC-7901	-	13.77	14.38	-	-	0.003	-
HT-29	-	-	-	-	-	0.004	-
P-388	1.73	0.71	0.0003	-	-	0.00008	-
U-251	-	-	-	-	-	0.005	-

Taxol and hydroxy-camptothecin (HCPT) are positive control drugs

## DISCUSSION AND CONCLUSION

Cancer is one of the most widespread diseases in humans and there is considerable scientific and commercial interest in the continuing discovery of new anticancer agents from natural product sources.[21,22] Currently, over 50% of drugs used in clinical trials for anticancer activity were isolated from natural sources or are related to them.[23] A number of active compounds have been shown to possess anticancer activity; these include flavonoids, diterpenoids, triterpenoids, and alkaloids.[17] Several mechanisms have been proposed to explain the cancer-preventive effects of plants. These include inhibition of mutagenesis by inhibiting the metabolism, inhibition of DNA adduct formation, free-radical scavenging, and effects on cell proliferation and tumor growth.[24] In the present study, the petroleum ether and ethylacetate extracts of ethanolic extract of *A. conyzoides* showed inhibitory activity on a wide range of cancer cell lines with ethylacetate extract having the greatest activity. It could therefore be deduced that the ethylacetate and petroleum ether extracts of *A. conyzoides* possess anticancer properties. Flavonoids have been implicated as responsible for the anticancer activities of some medicinal plants. The study of[17] showed that the flavonoids *cis*-2,5,7-trihydroxyflavanonol-3-O-β-D-glucopyranoside and chrysin-6-C-β-D-glucopyranosyl-8-C-α-L-arabinopyranoside exhibited significant cytotoxicity activity on A-549 and BGC-823 cancer cell lines. Thus, flavonoids may be responsible for the anticancer activity of *A. conyzoides*. Studies conducted by[25,26] reported a high correlation between DPPH radical scavenging potential and total phenolic content. Kaempferol (3,5,7,4′-tetrahydroxyflavone) is one of the most commonly found dietary flavonols. Kaempferol has also been found to be a blocker of reactive oxygen species (ROS) production by low K^+^-induced apoptosis in cerebella granule cells.[27] According to previous reports, antioxidant activity of kaempferol was believed to have cytoprotective function against oxidative stress. It seemed to suggest that kaempferol may not only protect cells from free radical damage via antioxidant effect, but also induce apoptotic cell death, via pro-oxidant activity, in malignant cell lines and to inhibit tumorigenesis.[28] The antitumor, anti-inflammatory, antiulcer activity, and HIV protease inhibitory activity of kaempferol have also been reported.[20,29] The result of the present investigation showed that *A. conyzoides* possesses antiradical activity and the property resides on the ethyl acetate extract. Kaempferol, which was isolated from this extract, was found to scavenge DPPH radical. It could therefore be deduced that the bioactive compound responsible for the antiradical activity of *A. conyzoides* is 3,5,7,4′-tetrahydroxyflavone (kaempferol). Further research is needed to unravel the specific bioactive compounds responsible for the anticancer properties of the extracts of *A. conyzoides*. In conclusion, the study has not only established the anticancer property of the extracts of *A. conyzoides* but also its antioxidant activity. Thus, the plant could be employed in ethno-medicine for the management of cancerous diseases.
